# Estimated impact of replacing sitting with standing at work on indicators of body composition: Cross-sectional and longitudinal findings using isotemporal substitution analysis on data from the Take a Stand! study

**DOI:** 10.1371/journal.pone.0198000

**Published:** 2018-06-13

**Authors:** Ida Høgstedt Danquah, Eva Sophie Lunde Pedersen, Christina Bjørk Petersen, Mette Aadahl, Andreas Holtermann, Janne S. Tolstrup

**Affiliations:** 1 National Institute of Public Health, University of Southern Denmark, Copenhagen, Denmark; 2 Research Centre for Prevention and Health, Glostrup University Hospital, Glostrup, Denmark; 3 Faculty of Health and Medical Sciences, Dept. of Public Health, University of Copenhagen, Copenhagen, Denmark; 4 National Research Centre for Working Environment, Copenhagen, Denmark; TNO, NETHERLANDS

## Abstract

The purpose was to examine and compare the effects of replacing time spent sitting with standing at work on fat-free mass, fat mass and waist circumference using isotemporal substitution. Analyses were conducted on work hours on both cross-sectional and longitudinal data. The study included 223 persons from an intervention study aimed at reducing sitting time at work among office employees. Sitting, standing and anthropometry were measured objectively. Cross-sectional isotemporal substitution analyses were modelled on baseline data, while longitudinal analyses were modelled based on differences in sitting and standing time at work between baseline and 1-month follow-up in relation to differences in anthropometric measures between baseline and 3-months follow-up. Replacing one hour of sitting time with one hour of standing was associated with a 0.21 kg higher fat-free mass in the longitudinal analysis and 0.95 kg in the cross-sectional analysis. Fat mass was 0.32 kg lower in the longitudinal analysis and 0.61 kg lower in the cross-sectional analysis. Waist circumference decreased by 0.38 cm in the longitudinal analysis and 0.81 cm in the cross-sectional analysis. Both cross-sectional and longitudinal analyses showed an effect on body composition measures by replacing one hour of sitting with standing however, this effect was largest in the cross-sectional analyses.

**Trial registration** ClinicalTrials.gov NCT01996176.

## Introduction

Isotemporal substitution analysis was developed within nutrition research, but in recent years it has been used in physical activity research to estimate the health effects of replacing one activity with another [1]. The idea is, as total time in a day is finite, activities are interdependent; more time spent in one activity results in less time spent in another. Cross-sectional studies have found that replacing sedentary time with either standing, light-intensity or moderate to vigorous physical activity was associated with lower body mass index (BMI), smaller waist circumference, improved cardio-metabolic risk biomarkers, higher insulin sensitivity, lower risk of depression and reduced mortality [2–11]. However, results based on cross-sectional data represent inter-individual comparisons estimating what would hypothetically happen if sitting was exchanged for some other activity while all other personal characteristics were kept constant. In contrast, a setup with repeated intra-individual measurements generates more valid estimates and therefore a better causal understanding of the effects of substituting one activity with another.

Results from the randomized controlled trial Take a Stand! showed a decrease in sitting time by more than one hour and a lower body fat percentage as an effect of the intervention [12]. In the present study we treated these data as a cohort study, pooling intervention and control groups. The aim was to compare the effects of replacing one hour of sitting with one hour of standing during work hours, as assessed by isotemporal substitution analysis on baseline (cross-sectional) and repeated measurement (longitudinal) data.

## Methods

### Study population

Take a Stand! was a cluster randomized controlled trial conducted in 19 offices (clusters) in 4 workplaces in Denmark and Greenland from November 2013 to June 2014. The aim was to reduce sitting time among office workers. Methods and results have been described in detail elsewhere [12]. In brief, workplaces were recruited through a press release and an open invitation in an electronic newsletter to both municipalities and private workplaces all over Denmark. Eligible workplaces were office-based with employees who sat for most of the workday. Participants were recruited through their workplaces and invited by e-mail to participate. Eligible participants were ≥18 years, understood Danish and worked >30 hours per week. Exclusion criteria were sickness or disabilities affecting the ability to stand or walk, and pregnancy. All participants had sit-stand desks. Participants were informed in writing and orally and signed informed consent forms. Sample size calculations are described in details elsewhere [12], but in brief they were based on an expected reduction in sitting time of 60 minutes. We assumed a standard deviation on daily sitting time of 100 min, intra-class correlation coefficient of 0.2, 80% power, a two-sided test and a significance level of 5%. This resulted in a required sample size of at least 300 participants from 12 offices. In total, 317 office employees participated.

The trial was approved by the local Ethics Committee in Denmark (H-6-2013-005) and in Greenland (project 20914–3, id: 2014–095402) and was registered at Clinicaltrials.gov (NCT01996176). Procedures were designed in accordance with the Helsinki Declaration.

### Assessments

Data was collected at baseline (anthropometry, activity measures and questionnaire data), after 1 month (activity measures) and after 3 months (anthropometry and activity measures). Questionnaire data was web-based and included information on work environment, socio-demographic factors, health status, and health behaviour.

### Activity measures

Participants wore an ActiGraph GT3X+ accelerometer, which records tri-axial accelerations and was set to record with 30 Hz [12]. The device is waterproof. It was taped to the front of the thigh, midway between the hip and knee joint, and was worn 24 hours a day for 5 consecutive days (Monday to Friday). The accelerometer was only removed in case of prolonged water activities (>30 min), contact sport or skin irritation. Participants kept a log during the accelerometer period, where they recorded time for sleep and work. Any irregularities, e.g. problems with the accelerometer and days off work, were also noted in the log.

Accelerometer data was processed using Acti4 software, specifically developed for thigh fixation of the ActiGraph [12]. Acti4 data processing has been validated in different settings and has high sensitivity and specificity to distinguish sitting from standing posture [13–15]. Activities were analysed with a minimum bout length of 5 sec for sitting and 2 sec for standing. Non-wear time was identified in 3 ways and data from those times was discarded, including a buffer of 10 minutes before and after: 1) If reported in the log; 2) if detected manually during data processing; or 3) if detected by Acti4 (a combination of >60 minutes with no movement immediately preceded by strong acceleration [14]). Time at work, leisure time and sleep were distinguished using log information. Only working hours were included and eligible workdays had to include at least 4 hours of work.

Time at work was divided into 3 categories: time spent sitting, standing and ‘other’. As only a fraction of total time at work was spent on others activities such as walking, running and biking (Table 1), time spent in all other activities than sitting and standing was combined into the ‘other’ category.

**Table 1:** Baseline characteristics of participants. N = 223.

	N (%)
**Demographic factors**	
Age (years, mean [SD])	47 (10.2)
Females	154 (69)
Married/living together	173 (78)
Tertiary education	157 (71)
**Health and health behaviour**	
BMI (mean [SD])	26.2 (4.6)
Body fat percentage	
Men	22.5 (7.1)
Women	34.0 (7.7)
Fat-free mass (kg)	
Men	66.9 (6.4)
Women	46.6 (5.1)
Fat mass (kg)	
Men	20.4 (9.0)
Women	25.5 (10.6)
Waist circumference (cm)	
Men	98.6 (12.3)
Women	88.1 (13.0)
Smokers	28 (13)
Self-rated health	
Excellent/very good	75 (34)
Good	127 (57)
Less good/bad	19 (9)
**Activity variables**	**Mean (SD)**
Workplace sitting, h/8h workday	5.7 (0.95)
Workplace standing, h/8h workday	1.5 (0.81)
Workplace other, h/8h workday	0.84 (0.29)
Leisure time sitting, h/8h leisure	4.89 (0.85)
Leisure time MVPA, h/8h leisure	0.72 (0.33)

MVPA–Moderate to vigorous physical activity; SD–Standard Deviation

### Anthropometric outcomes

Fat mass and fat-free mass were measured with bioimpedance to the nearest 0.1 kg using BC-418 MA, Tanita Corp., Tokyo, Japan. Waist circumference was measured midway between the lower rib and the iliac crest to the nearest 0.1 cm with a non-stretchable measuring tape by the same observer at baseline and at 3-months follow-up.

### Statistical analyses

Participants with valid anthropometric measures, who had at least one day of accelerometer measurements, were included in the analyses. Of 317 participants, 38 were lost to the 1-month follow-up, 50 were lost to the 3-months follow-up, and 6 participants had missing information on body composition, leaving 223 for analyses. The study population was the same in both longitudinal and cross-sectional analyses in order to enhance comparison of the results.

Isotemporal substitution analyses were conducted to estimate the effects of replacing 1 hour of sitting with standing on fat-free mass, fat mass and waist circumference. Total time was held constant by standardising time at work to 8 hours. Time spent sitting and standing was calculated for each individual as the average over the number of valid accelerometer days (1–5 days) at baseline and follow-up. For the longitudinal analyses, in order to ensure that changes took place before assessment of the outcome, we calculated changes as the differences in time spent sitting and standing between baseline and 1-month follow-up, and examined the effect of these differences on differences in anthropometric measures between baseline and 3-months follow-up.

Multilevel linear regression modelling was used for the isotemporal substitution analyses. Because time at work was standardised to 8 hours the sum of changes in time spent sitting, standing and on other activities at work between baseline and 1-month follow-up equals zero (Δ sit_(baseline→1mo follow-up)_ + Δ stand_(baseline→1mo follow-up)_ + Δ other_(baseline→1mo follow-up)_ = 0), the same applies to differences at baseline for the cross-sectional analyses. To quantify the effect of replacing sitting with standing, sitting was removed from the model and the estimates of the two remaining activities were interpreted as the effects of replacing 1 hour of sitting with standing or other activities. All models were adjusted for age, sex and BMI at baseline. Thus, for example for the longitudinal analyses, when estimating the effect of replacing one hour of sitting with 1 hour of standing on fat-free mass, the equation for the statistical model was:

$$\begin{array}{l} \Delta_{\left(baseline\to 3mo\;follow-up\right)}\mu_{ijk}\\ \;=\alpha +\beta 1*{\Delta stand}_{\left(baseline\to 1mo\;follow-up\right)}+\beta 2*{\Delta other}_{\left(baseline\to 1mo\;follow-up\right)}\\ \;+\beta 3*age+\beta 4*sex+\beta 5*BMI+\beta 6*workplace+{\gamma office}_{\left(workplace\right)} \end{array}$$

where Δ_((baseline→3mo follow-up)_μ_ijk_ is the difference in fat-free mass between baseline and 3-months follow-up for person *i* in office *j* at workplace *k*.

For cross-sectional analyses the following model was used to analyse the association between differences at baseline and anthropometric characteristics at baseline:

$$\mu_{ijk}=\alpha +\beta 1*{stand}_{\left(baseline\right)}+\beta 2*{other}_{\left(baseline\right)}+\beta 3*age+\beta 4*sex+\beta 5*BMI+\beta 6*workplace+{\gamma office}_{\left(workplace\right)}$$

where μ_ijk_ is the fat-free mass at baseline for person *i* in office *j* at workplace *k*.

The models assume linear relationship between independent and dependent variables which was determined by examining plots showing residuals versus predicted values of dependent and independent variables prior to running the analyses. Normal probability plots of residuals showed acceptable fit of the final linear regression models.

Analyses were conducted using STATA/IC-14.0.

## Results

The mean age of the 223 participants was 47, the majority were women (69%), and the mean sitting time at work was 5.7 h per 8 h workday at baseline (Table 1).

The median wear time of the ActiGraph was 4.0 days (interquartile range: 4.0–5.0) at baseline and 4.5 days (interquartile range: 4.0–5.0) at 1-month follow-up.

**Fig 1:**
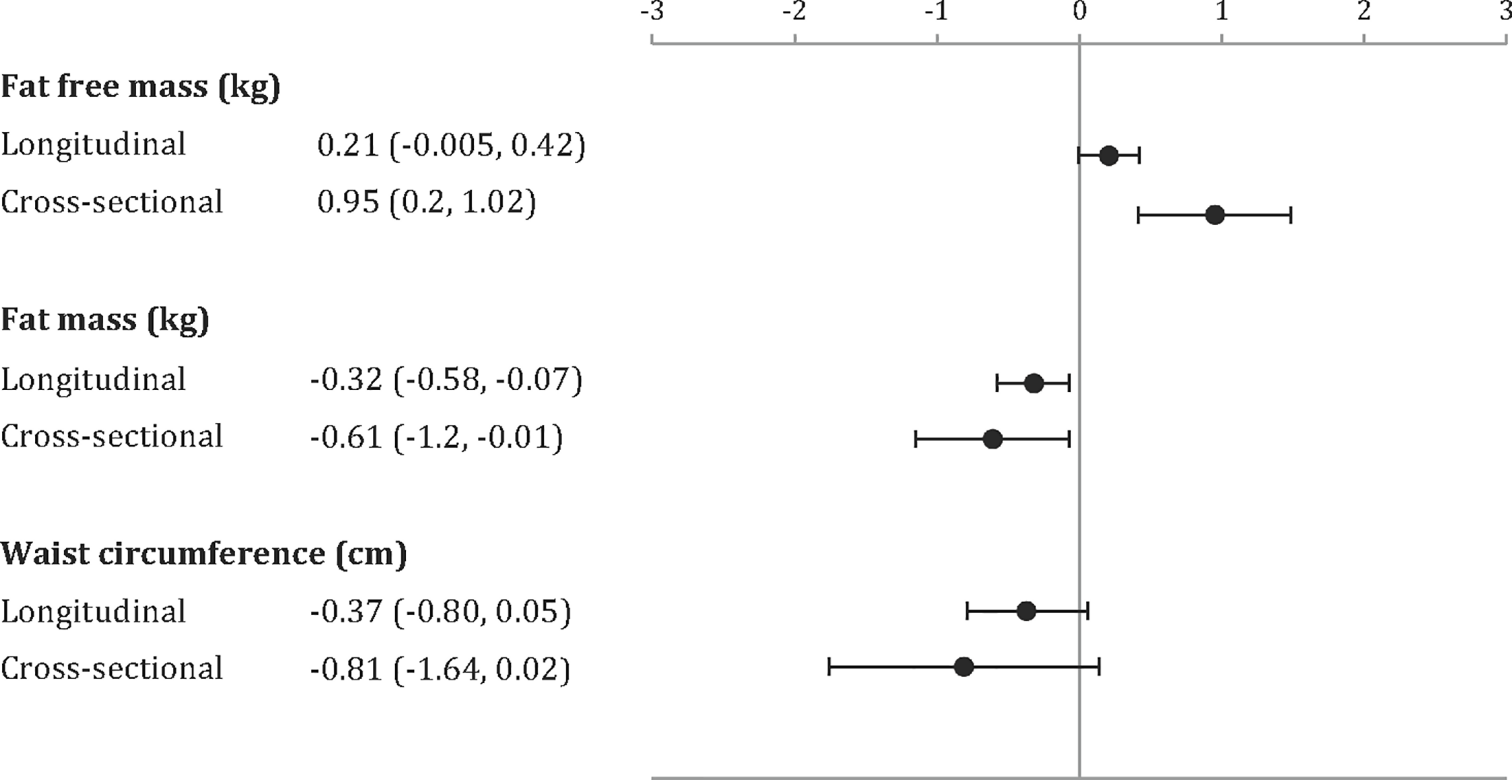
Isotemporal substitution models showing effect of replacing 1 hour of sitting with 1 hour of standing on changes in fat-free mass, fat mass and waist circumference from longitudinal and cross-sectional analyses.

In longitudinal analyses, replacing 1 hour of sitting time with 1 hour of standing was associated with an increase in fat-free mass by 0.21 kg (95% confidence limits (CL): 0.00, 0.42), a decrease in fat mass by -0.32 kg (95% CL: -0.58, -0.07) and a decrease in waist circumference by -0.37 cm (95% CL: -0.80, 0.05) (Fig 1).

For cross-sectional analyses, replacing 1 hour of sitting with 1 hour of standing was associated with 0.95 kg (95% CL: 0.2, 1.02) higher fat-free mass, -0.61 kg (95% CL: -1.2, -0.01) lower fat mass and -0.81 cm (95% CL: -1.64, 0.02) lower waist circumference (Fig 1).

## Discussion

Our results demonstrated that replacing 1 hour of sitting with standing resulted in changes in body composition measures. These changes were 2 to 4 times as great when analysing cross-sectional data compared to the longitudinal data results. For example, for fat-free mass, replacing 1 hour of sitting with standing resulted in an increase of 0.95 kg in the cross-sectional data and 0.21 kg in the longitudinal data.

To the best of our knowledge, this is the first study to examine the effects of replacing sedentary time at work on anthropometric measures by comparing cross-sectional and longitudinal data in isotemporal substitution analysis. To date, studies examining the replacement associations of sitting time and other behaviours with health parameters have mainly studied activities cross-sectionally and thus made use of inter-individual comparisons.

Results from the cross-sectional data showed that replacing 1 hour of sitting with 1 hour of standing was associated with a difference in waist circumference of -0.81 cm. Van Der Berg et al. found that replacing 30 min sitting with standing resulted in a difference in waist circumference of -0.41 cm, which is similar to our finding for 1 hour’s change [10], while Healy et al. in their study found no effect on waist circumference when replacing 2 hours of sitting with standing; however, they found an effect on blood biomarkers [7].

Other studies have used data from waist-worn accelerometers, which are not able to distinguish sitting from standing. For example Healy et al. looked at the effects of replacing 30 minutes of prolonged sedentary time with light activity and found a change in waist circumference of -0.77 cm [6]. Compared to our results for 1 hour replacement this change is bigger; however, this is expected as replacing sedentary time with light activity might cause a bigger change in energy expenditure than replacing sitting with standing. Buman et al. found a 2.8% decrease in waist circumference from replacing 30 minutes of sedentary behaviour with moderate to vigorous physical activity and no changes when replacing sedentary behaviour with light physical activity [2]. In our study, a 2.8% decrease would be equivalent to 2.5–2.8 cm for a 30-minute change, and thus a greater decrease than we found, which is predictable, as replacing sedentary time with physical activity causes higher energy expenditure than replacing sitting with standing. No other studies have looked at fat mass or fat-free mass.

In the present study, use of cross-sectional data resulted in effects of greater magnitude than analyses of longitudinal data. A reason for this could be that effects are overestimated in the cross-sectional analyses due to residual confounding, i.e. differences between participants such as dietary patterns or health variables not accounted for. Another explanation is reverse causation: when looking at cross-sectional data it is not possible to determine e.g. whether some people are sitting less because they have high waist circumference or if they have high waist circumference because of they do not sit much. Results from the analyses of the longitudinal data are also subject to discussion especially due to the limited time frame; 3 months may not reflect changes over a longer period of for instance years. In long periods, higher effects of replacing sitting with standing might be observed.

Isotemporal substitution analysis is just one way of modelling data. Another relevant method suitable for analysing time substitution is compositional data analysis [16].

A key strength of the study is the possibility of comparing cross-sectional and longitudinal data from the exact same participants. Our study involved a relatively large number of participants, and 76% of eligible individuals participated. Moreover, we used objective, validated methods to estimate both exposure and outcome measures.

Limitations of the study include lack of generalisability because the study sample only included office workers. However, office workers constitute a rather large group and replacing sitting with standing is highly relevant in this group.

Activity measures were obtained for 5 days only (Monday to Friday) and may therefore not represent true habitual behaviour. However, in another study we have looked at intra-individual variability in day-to-day measurement of sedentary behaviour and found that 4.2 to 4.7 measurement days were adequate to estimate time spent sitting and standing during working hours, as the variation on workdays is low [17].

Another limitation of the study is that measurements of fat mass and fat-free mass were obtained using bioimpedance, as used by Tanita BC-418 MA, which is subject to measurement error. A study comparing Tanita BC-418 MA with the often considered gold standard, DEXA-scanning (Dual Energy X-ray Absorptiometry) showed that Tanita BC418 MA underestimated body fat percentage by 3–5% compared to DEXA [18]. However, this error is likely to be random.

Finally, studies examining the effect of replacing sitting with standing on energy expenditure have found a very small increase in energy expenditure from increased standing [19, 20]. It is thus plausible to expect other factors such as diet to influence the results. However, no information on food intake was collected and therefore it is not possible to tell if changes or differences in energy intake could explain any of our findings. It might be that participants who changed their sitting or standing behaviour also changed their dietary pattern, in turn affecting body composition.

## Conclusions

Replacing sitting with standing during working hours resulted in small but significant changes in fat-free mass, fat mass and waist circumference. Thus even small changes during working hours could have an effect on health. We also found that cross-sectional analyses resulted in more than twice the effects on body composition measures compared to longitudinal analyses. This may be due to real differences stemming from e.g. difference in time frames. Alternatively, results from cross-sectional analyses, reflecting inter-individual comparisons are subject to residual confounding from associated risk factors.
